# Genetic risk score as a predictor of gestational diabetes in Central European Caucasians

**DOI:** 10.1038/s41598-026-49602-z

**Published:** 2026-04-29

**Authors:** Jaroslav A. Hubáček, David Galuška, Beáta Krejčířová, Vendula Bartáková, Katarína Chalásová, Lukáš Pácal, Věra Lánská, Petr Janků, Veronika Ťápalová, Kateřina Kaňková

**Affiliations:** 1 Experimental Medicine Centre, Institute for Clinical and Experimental Medicine, 14021 Prague 4, Czech Republic; 2 3rd Department of Internal Medicine, 1st Faculty of Medicine, Charles University, Prague, Czech Republic; 3 Department of Pathophysiology, Faculty of Medicine, Masaryk University, Brno, Czech Republic; 4 Department of Obstetrics and Gynaecology, University Hospital Brno, Brno, Czech Republic; 5 Department of Health Sciences, Faculty of Medicine, Masaryk University, Brno, Czech Republic

**Keywords:** Gestational diabetes, Polymorphism, Genetic risk score, Type 2 diabetes, Genetics, Molecular biology, Molecular medicine

## Abstract

Gestational diabetes mellitus (GDM) is a common complication of pregnancy associated with perinatal complications and increased future risk of T2DM. GDM has a polygenic background partly overlapping with T2DM, and using a genetic risk score (GRS) that captures complex genetics may improve early detection of individuals at risk. SNPs previously associated with T2DM in the Czech population (21 loci) have been examined in a study comprising Caucasian (i) pregnant women with GDM (N = 416), (ii) non-diabetic population controls (N = 1170) and (iii) T2DM patients (N = 359) to analyse (di)similarities of T2DM and GDM genetic architecture using both weighted (wGRS) and unweighted (uGRS) GRS constructed based on single locus analyses. Fourteen of 21 SNPs had no influence on the GDM development. The most significant SNPs associated with GDM were within the *ARAP1* (rs1552224; OR, 95% CI 2.98, 2.18–4.08) and *MNTR1B* (rs10830963; OR, 95% CI 1.78, 1.41–2.24) genes. Both uGRS (*P* < 0.0001) and wGRS (*P* < 0.0001), comprising alleles of the seven most strongly associated variants, were associated with GDM, with wGRS being a better discriminator—AUC 0.669 vs. 0.628 (*P* < 0.001). Carriers of the population-derived Q5 of wGRS (in comparison with Q1) have a significantly increased risk of GDM with OR (95% CI) 6.8 (4.3–10.9); *P* < 1.10^−6^. The same scores derived from the *ARAP1*, *NOTCH2*, and *MTNR1B* genes discriminate between the GDM and T2DM groups (*P* < 0.0001). Identification of statistically significant association of the population-specific wGRS with GDM susceptibility supports its potential clinical utility for the risk stratification of pregnant women (beyond established non-genetic risk factors). Yet, the actual health care adoption of GRS in the near future is unlikely, since additional validation, regulatory and administrative steps, thorough economic analyses, ethical discussion, etc. are certainly required.

**Supplementary Information:**

The online version contains supplementary material available at 10.1038/s41598-026-49602-z.

## Introduction

Pregnancy is accompanied by progressive insulin resistance. Dysregulation of this process can lead to the development of gestational diabetes mellitus (GDM). GDM affects 10–15% of pregnant European women and is defined as hyperglycaemia that does not reach the threshold for overt diabetes outside pregnancy^1^. GDM usually resolves after delivery, but it may have two important health consequences. In the short term, GDM increases the risk of complications during pregnancy (e.g. gestational hypertension or preeclampsia) as well as during delivery (e.g. macrosomia). In the long term, GDM increases the risk of type 2 diabetes mellitus (T2DM) and cardiovascular disease^2^. The main risk factors for the development of GDM are overweight/obesity, physical inactivity, smoking, increased age at pregnancy, ethnicity (non-white ancestry) and family history of T2DM or GDM^1,3^. The latter factor suggests that a significant proportion of GDM risk is mediated by the genetic background that may share similarities with T2DM.

In contrast to the large number of genome-wide association studies (GWAS) focusing on T2DM [for example,^4–6^, for review see^7^], the number of GWAS attempting to dissect the genetics of GDM is much more sparse^8–10^, and only a subset of these studies included Caucasian populations. The most significant variants associated with GDM have been located at the *MTNR1B*, *TCF7L2* and *CDKAL1* loci^10^, and these genes have also been identified as important genetic determinants of T2DM^4–6^.

GDM is a polygenic disease^1^ where the overall degree of genetic risk/susceptibility can be assessed using a genetic risk score (GRS) calculated from the number of risk alleles present in a given individual^11,12^. Unweighted GRSs (uGRS) simply summarise the number of risk alleles in each subject, without considering the absolute effect of each allele. Weighted GRS (wGRS) consider the odds or hazard ratios identified for each risk allele or genotype. To date, studies utilising the GRS approach to assess GDM genetic architecture in Central European populations are lacking.

Based on the known facts mentioned above, we hypothesise that the genetic architecture of GDM partly overlaps with that of T2DM, and GRS constructed using T2DM GWAS-identified variants (conferring a maximal risk in the Czech population based on previous studies) might successfully identify GDM subjects at risk even before their diagnosis in the 2^nd^ trimester. The selection of particular SNPs of interest was based on previous analyses in T2DM and GDM of European ancestry (10) or the Czech population specifically^13^.

The aims of the study were i/ to analyse the 21 preselected SNPs potentially associated with GDM, and ii/ based on single-locus analyses, to create a specific GRS that shall help to distinguish between GDM, controls and T2DM (Table 1).

**Table 1:** Characteristics of study participants.

Parameter	GDM	Controls	T2DM
N	416	1.170	359
Age (years)	33 [30–36]	48 [40–56]	62 [50–75]
BMI (kg/m^2^)	26.1 [22.6–30.8]	26.2 [23.3–30.1]	28.7
Obesity	28.6	25.6	32.5
Smokers	25	23.4	27.3
Hypertension	4.4	17.5	73.1
FPG (mmol/l)	5.1 [4.6–5.4]	n.a	n.a
2 h post oGTT glyceamia (mmol/l)	8.7 [7.9–9.3]	n.a	n.a
Triacylglycerols (mmol/l)	3.45 [2.75–4.2]	1.23 [0.91–1.65]	n.a
Weight gain (kg)	9 [6–11]	n.a	n.a
Pregnancy after IVF	9.0	n.a	n.a
Comorbidities, any	63.4	n.a	n.a
Polycystic ovary syndrome	1.0	n.a	n.a
Preeclampsia	10.6	n.a	n.a
Thyroid disorders	15.0	n.a	n.a
Other autoimmune disease	3.0	n.a	n.a
Coagulation disorder	4.1	n.a	n.a
Anaemia	10.6	n.a	n.a
Allergy or asthma	29.5	n.a	n.a
Other comorbidity	26.7	n.a	n.a
DM in family	67.4	n.a	n.a
DM duration (years)	n.a	n.a	13 [7–26]

Continuous parameters are presented as mean [IQR], prevalence of other characteristics is in %

n.a., not analysed.

(GDM, gestational diabetes mellitus; T2DM, type 2 diabetes mellitus; BMI, body mass index; FPG, fasting plasma glucose; oGTT, oral glucose tolerance test; IVF, in vitro fertilisation; DM, diabetes mellitus).

## Material and methods

### Study subjects

The case–control study included three independent cohorts for mutual comparisons among GDM, T2DM and non-diabetics. A total of 416 pregnant women with GDM were recruited between 2015 and 2019 using following criteria: (i) inclusion criteria—positive screening for GDM by glucose tolerance test (oGTT) at mid-gestation (see below), singleton pregnancy, and Caucasian origin, (ii) exclusion criteria—pre-existing T1DM or T2DM or diabetes with established treatment before pregnancy, non-Caucasian origin and multiple pregnancies. Diagnosis of GDM was based on the results of a routine oGTT performed between the 24^th^ and 28^th^ week of pregnancy according to the IADPSG criteria: fasting plasma glucose (FPG) ≥ 5.1 mmol/l, 1-h post-load glucose ≥ 10 mmol/l and 2-h post-load glucose ≥ 8.5 mmol/l (any one of the three values above cut-off fulfilled the diagnosis of GDM). Enrolled subjects were followed in the Diabetes Centre of the Faculty Hospital Brno until delivery in the Dept. of Gynaecology and Obstetrics of the same hospital.

The T2DM group comprised 359 females followed at the Diabetology Clinic of the Institute for Clinical and Experimental Medicine (Prague) between 01/2012 and 04/2021; for more details on subjects, see Hubacek et al.^13^.

The control group comprised 1170 women selected from the post-MONICA study, a sample of the general population aged 26–65 years at the time of enrolment^14^. All control subjects had at least one child of their own, did not self-report any type of diabetes and were not prescribed antidiabetic treatment.

All participating subjects were of Caucasian origin.

### Genotyping and genotyping quality control

DNA was extracted from EDTA anticoagulated peripheral blood using the phenol–chloroform method, salting-out method or commercial kit (QIAamp DNA Blood Mini Kit, Ref 51,106) and stored at −20 °C until analysis. Individual SNPs (listed in detail in Suppl. Table 1) have been genotyped using TaqMan assays or the PCR-RFPL method [for more details see^13^^,^^15^]. To ensure genotyping accuracy, 100 DNA samples have been independently genotyped at both participating laboratories (at the Institute for Clinical and Experimental Medicine in Prague and the Department of Pathophysiology, Faculty of Medicine of Masaryk University) with 100% accuracy.

### Single locus analyses, statistical analysis and construction of GRSs

All subjects enrolled in the study had a complete set of 21 SNPs of interest successfully genotyped (no imputation has been performed). This represents 98.6% of GDM subjects, 96.9% of controls and, finally, 97.6% of T2DM patients from the originally collected and screened set of samples. Genotype frequencies of all determined SNPs were compared individually between the groups using the chi-square test in 2 × 3 tables. When fewer than 20 subjects are present in at least one of the groups being compared, a 2 × 2 table has been used. Initially, GDM subjects were compared with population controls and SNPs that were at least marginally (*P* < 0.10) associated with GDM in at least one model (dominant, codominant or recessive) (N = 9) were preselected to construct weighted and unweighted GRS. For two genes (KCNJ11 and WFS1), differences between groups have been primarily driven by differences in the prevalence of heterozygotes. These SNPs were subsequently omitted from the GRS calculations as the differences likely represent the false positive findings. Variants within seven genes (*TCF7L2*, *IRS1*, *CDKAL1*, *ARAP1*, *NOTCH2*, *FTO* and *MNTR1B*) were therefore selected for further analysis using GRS for risk of GDM and variants within three genes (*ARAP1*, *NOTCH2*, and *MNTR1B*) for subsequent indirect analysis of T2DM risk in GDM subjects. The unweighted GRS (uGRS, presented as mean ± SD) was calculated by summing the T2DM risk alleles (homozygote for risk allele contributed 2 points, heterozygote 1 point and 0 points for the subject without risk allele). Weighted GRS (wGRS) has been calculated as log of ORs ratios, based on the results presented in Table 2.

**Table 2:** Individual variants significantly associated with GDM used for the construction of specific GRS.

Gene	SNP	Calculated for	OR (95% CI)^#^	P^#^	OR (95% CI)^&^	P^&^	OR (95% CI)^±^	P^±^
*TCF7L2*	rs7903146	+ T vs. CC	1.34 (1.07–1.67)	0.011	0.90 (0.67–1.20)	0.452	1.49 (1.17–1.89)	0.001
*IRS1*	rs2943640	CC vs. + A	1.45 (1.16–1.82)	0.001	1.30 (0.98–1.73)	0.072	1.12 (0.88–1.42)	0.371
*CDKAL1*	rs7756992	+ G vs. AA	1.29 (1.03–1.62)	0.025	1.06 (0.80–1.41)	0.662	1.21 (0.96–1.54)	0.108
*ARAP1*	rs1552224	AA vs. + C	2.98 (2.18–4.08)	0.000	1.93 (1.32–2.83)	0.001	1.54 (1.17–2.04)	0.002
*NOTCH2*	rs10923931	+ T vs. GG	1.30 (1.00–1.68)	0.046	1.52 (1.08–2.15)	0.014	0.85 (0.83–1.14)	0.281
*MNTR1B*	rs10830963	+ G vs. CC	1.78 (1.41–2.24)	0.000	1.60 (1.20–2.13)	0.001	1.11 (0.88–1.41)	0.377
*FTO*	rs9936385	CC vs. + T	1.29 (0.99–1.69)	0.066	0.87 (0.63–1.21)	0.409	1.48 (1.12–1.95)	0.004

^#^Control females vs. GDM.

^&^GDM vs. T2DM females.

^±^T2DM females vs. control females.

(GDM, gestational diabetes mellitus; T2DM, type 2 diabetes mellitus; GRS, genetic risk score).

For graphical presentation and comparison of wGRS, quintiles (Q1–Q5) have been created, based on the number of subjects in the population controls.

Areas under the ROC curve (AUC/ROC) were compared by the deLong paired test. Comparison of GRS between groups was done by the Wilcoxon test. JMP15.2.0, SAS Institute Inc. 2019 software platform has been used for statistical analyses. *P* values < 0.05 were considered significant.

## Results

General characteristics of the examined cohorts are shown in Table 1.

### Effect of individual SNPs

Genotype and allele frequencies within all three examined groups are summarised in detail in Supplementary Table 2.

Twelve SNPs showed an almost identical distribution of genotypes and alleles across all three examined groups; for two additional SNPs (*KCNJ11* and *WFS1*), differences between groups were mainly driven by heterozygotes.

We identified an association between 7 SNPs and GDM susceptibility, namely *TCF7L2*, *IRS1*, *CDKAL1*, *ARAP1*, *NOTCH2*, *FTO* and *MNTR1B* (details are provided in Table 2). Variants most strongly associated with GDM were localised within the *ARAP1* and *MNTR1B* genes (both *P* < 0.0001). Notably, these two genes (together with *NOTCH2* and *TCF7L2*) have also been able to discriminate between GDM and T2DM in our subgroup analysis (see Suppl. Tab. 2 for P values).

Finally, the genotype frequencies of four genes (*IRS1*, *CDKAL1*, *NOTCH2,* but not *MTNR1B*) did not differ between population and T2DM females (see Table 2), suggesting that they do not represent the strong shared genetic susceptibility to both types of diabetes in the Czech population.

### Impact of GRSs on the GDM development

**Fig. 1:**
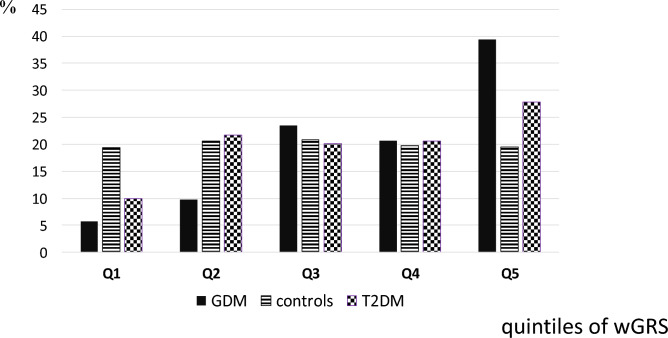
Distribution of weighted genetic risk score (wGRS) in examined groups. Due to the high number of individual categories (N = 277) quintiles (Q1–Q5) have been created according to the numbers of categories.

**Fig. 2:**
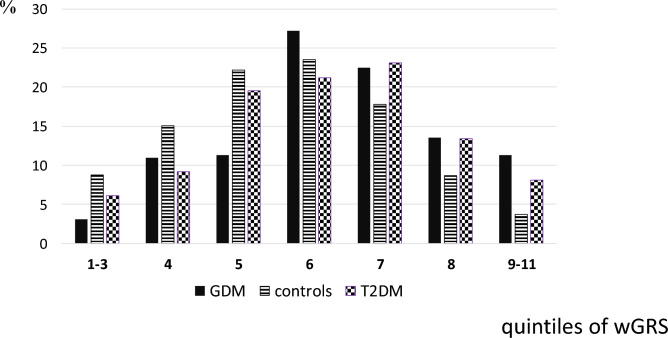
Distribution of unweighted genetic risk score (uGRS) in all examined groups.

**Fig. 3:**
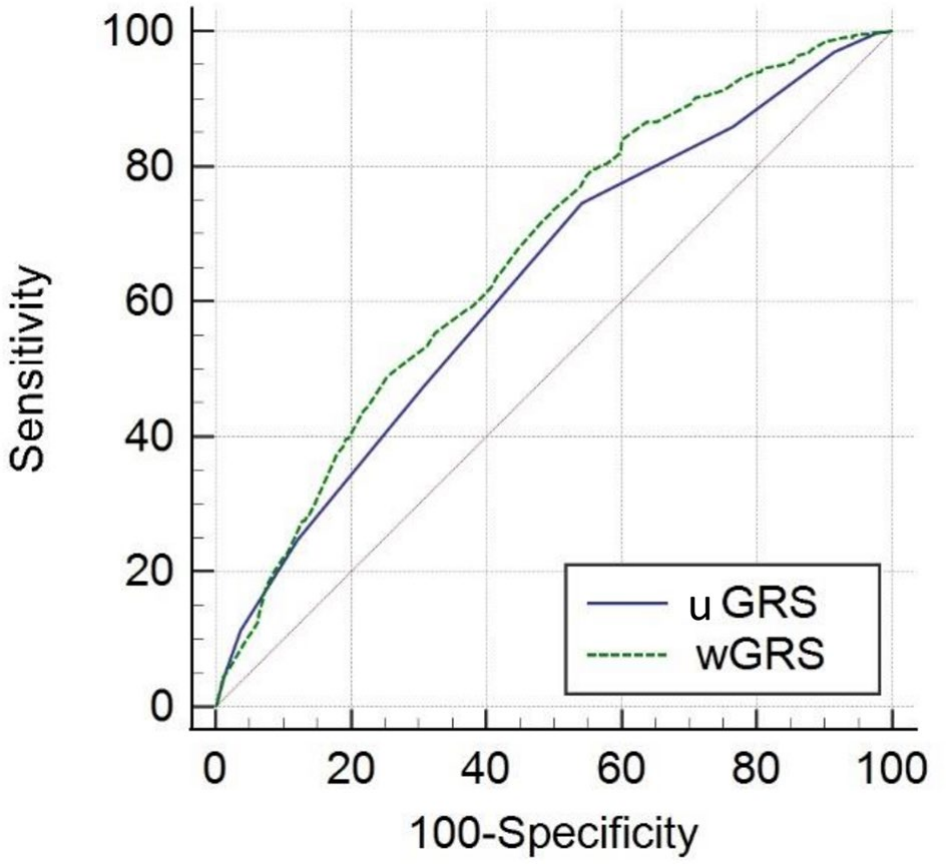
Comparison of AUC curves (GDM vs. controls) for wGRS and uGRS.

Both uGRS as well as wGRS have been associated with the increased risk of developing GDM. The mean uGRS was 6.54 ± 1.67 in the GDM group and 5.67 ± 1.61 in controls (*P* < 0.0001, Wilcoxon test); wGRS median (25% and 75% IQR) was 0.85 (0.71–0.99) and 0.72 (0.47–0.87), respectively (*P* < 0.0001, Wilcoxon test). Distributions of weighted and unweighted scores in the examined groups are shown in Figs. 1 and 2, respectively. Means of both scores were significantly higher in women with GDM than in controls (both *P* < 0.0001), but wGRS appeared to be a slightly better predictor of GDM in comparison with uGRS (AUC 0.669 vs. 0.628; *P* < 0.001; Fig. 3). Cross-validation was performed on three random subsets of the analysed data (presented in Suppl. Table 3); the predictive power of the models was assessed on subsets not included in their estimates using ROC analysis. The results are consistent across all three models (AUC = 0.653, 0.660, 0.661) and with the overall model (AUC = 0.669).

Ascertained AUC values are relatively modest in terms of rigorous predictive accuracy, and their potential current clinical relevance is limited at present. Yet, this is one of the first attempts to apply GRS to GDM genetics, and, obviously, more independent studies and replications are needed to bring the parameter into diagnostic use in the future.

Comparison of quintiles of wGRS, namely Q5 vs. Q1 revealed increased risk of GDM with OR (95%CI) 6.8 (4.3–10.9); P < 0.000001 (for more details see Fig. 1) for subjects with wGRS within Q5.

### Discrimination between GDM and T2DM subjects

Individual SNPs could be used for distinguishing between T2DM and GDM subsets, but, as the numbers are nominally lower, the differences have reached statistical significance only in the case of *ARAP1*, *NOTCH2* and *MTNR1B* (all *P* < 0.01, for more details see Suppl. Table 2). Interestingly, the risk associated with individual alleles is higher for the determination of GDM than for the determination of T2DM. Distribution of GDM risk alleles is significantly different (*P* < 0.0001) between the T2DM group and GDM subjects (Suppl. Figure S1), and also mean wGRS was significantly lower (*P* < 0.01) in T2DM subjects (0.52 ± 0.23) than in GDM (0.60 ± 0.20).

## Discussion

Screening at the mid-term of pregnancy for early identification of subjects at GDM risk could provide significant healthcare benefits, such as earlier and stricter treatment, and personalised pregnancy and postpartum follow-up. During the physiological pregnancy, placental hormone-mediated increase in insulin resistance in the second half of the pregnancy is paralleled by increased insulin secretion to maintain normoglycaemia and to provide a powerful pro-growth stimulus to the foetus by insulin^2^. However, women who are unable to increase insulin production sufficiently will develop a disturbance in glucose metabolism of varying severity. Both insulin resistance and some degree of impaired insulin secretion are typical abnormalities present in patients with T2DM, implying a common pathogenic mechanism and suggesting that GDM might be the first manifestation of T2DM (at least in some cases) rather than a unique pathophysiological entity. Unsurprisingly, many GDM genetic studies assume an overlap with T2DM in the genetic makeup of individuals, and there are studies confirming shared genetic backgrounds^3,16^.

Our current study, which focused on the genetic determination of GDM using GRS, is the first one in Central European subjects. The major findings can be summarised as follows: (i) seven of the 21 SNPs analysed have been associated with GDM in our population. Additionally, (ii) both uGRS and wGRS comprising these 7 SNPs were able to discriminate between GDM and control subjects significantly. Finally, (iii) indirectly, we suppose that a subset of three variants could distinguish between GDM and T2DM. Two of these polymorphisms (within *MTNR1B* and *NOTCH2*) were not significantly associated with T2DM in the Czech population^13^. Unfortunately, it is unclear what pathophysiological mechanism underlies these observations, and further detailed studies are necessary to confirm and explain the observed differences. Only the *ARAP1* variant revealed a gradual increase in risk from controls through T2DM to GDM. If these variants have the potential to help identify also the subjects with an increased risk of T2DM when GDM precedes, this needs to be resolved in a study with a different design.

As expected, one of the most significant determinants of GDM in our study was the *MNTR1B* gene that encodes for melatonin receptor 1B. This gene is strongly associated with chronotypes, and our results agree with the widely recognised function of this gene/protein in the determination of both GDM and T2DM [for review, see^17^].

By contrast, *ARAP1* (also known as *CENTD2*), a protein associated with the Golgi apparatus, where it plays a key role in insulin exocytosis, has received less attention in relation to determining GDM. In fact, this is the first study describing such a type of association in Caucasians. In other ethnic groups, the results are rather controversial. The *ARAP1* T2DM-associated allele increased the risk of GDM in Chinese^18^, but not in Mexican females^19^. Conversely, the same ARAP1 allele has been associated with a decreased risk of GDM in Indian females^20^. These differences may be a consequence of the ethnic differences between the studies. Therefore, our findings need to be replicated in another independent set of Caucasian GDM subjects.

Most of the studies focusing on GDM have, until recently, investigated selected individual polymorphisms originally associated with T2DM. A recent meta-analysis^21^ included 23 case–control genetic association studies and identified several variants across different ethnicities that were significantly associated with GDM. Of these, *MTNR1B*, *CDKAL1* and *TCF7L2* loci overlap with the findings from our study. It is interesting to note that the overlap of SNPs significantly associated with GDM is high, yet there are significant differences between ethnicities (see, for example, the aforementioned *ARAP1* gene results).

Using genetic risk scores to analyse the simultaneous, cumulative effects of several SNPs in a semi-continuous manner is the logical next step in deciphering the genetics of complex diseases. One of the first GRS studies^22^ that addressed the role of T2DM-associated variants and the risk of GDM used 11 variants and confirmed the significant role of four (within *FTO*, *TCF2*, *CDKAL1*, and *TCF7L2*) and a non-significant trend for the remaining six. In addition, uGRS exhibited a significant increase in GDM risk with an OR of 1.18 for each allele. A similar increase per allele (1.11) has been found in a Swedish GDM sample^23^, despite the fact that only two out of 11 SNPs (partially overlapping with the study conducted by Lauenborg et al.^22^) used for GRS calculation reached nominally significant differences. The set of 11 SNPs has been analysed in Indian females^20^, and an identical OR increase per allele of 1.11 has been found. Ding et al.^24^ determined 112 T2DM risk variants from the GWAS, identified 8 SNPs associated with GDM and confirmed 3 previously associated SNPs in their cohort. Using 11 SNPs again, they constructed a wGRS and found it associated with GDM. In a small Canadian study, the GRS derived from 36 SNPs (originally associated with T2DM) was also associated with GDM and with an increased risk of future T2DM^25^. Finally, clustering analysis^26^ of more than 200 SNPs across three cohorts (Gen3G, HAPO, and MGH^2^) revealed that two of five T2DM risk-variant clusters were also associated with GDM. These clusters were those that reduce β-cell function and cause abnormal hepatic lipid metabolism. Thus, the GRS has certainly improved our understanding of the role of genetic factors in GDM development; however, population-specific differences may halt progress toward more routine use of the GRS. Furthermore, the predictive power of GRS could be significantly modulated by the interplay with lifestyle factors, namely dietary habits, as recently shown^27^.

Not only the DNA sequence but also its epigenetic modifications may be important in GDM determination. For example, three differentially methylated positions were associated with GDM status with outstanding AUCs above 0.95^28^. Interestingly, the shared genetic architecture of T2DM and GDM has also been confirmed thanks to this approach—six different CpG sites clearly distinguished between controls and both GDM as well as T2DM^29^. The potential importance of DNA methylation in determining GDM and T2DM is further supported by the fact that *FTO*, one of the major genetic determinants of both T2DM and GDM, is an epigenetic modulator encoding a nucleic acid demethylase^30^.

We are aware of several limitations of our study. First, we do not have access to the independent cohorts for a replication study within the same population, which is crucial for eventual consideration of the GRS as a stratification tool. Furthermore, we unfortunately lack the information on the age and BMI values at the time of pregnancy in controls, and these variables could not be used to adjust our results for these two important non-genetic risk factors of GDM in the historical cohort. Finally, as in other studies, the selection of SNPs was based on findings obtained through screening of SNPs associated with T2DM. This approach could introduce some bias, meaning some SNPs that are exclusively associated with GDM may remain undetected. On the other hand, there are several strengths in our study. The comparison between three independent study groups—T2DM, GDM and healthy controls—allows us to ascertain differences or, conversely, a shared genetic background between groups. The differences obtained are robust, and the possibility of false negative results is low. Importantly, we have identified three variants, potentially able to discriminate between the GDM subjects with increased risk of T2DM. Finally, and importantly, our study is the first to have been performed on subjects from the Central European region, thus filling a gap in the general knowledge of the genetic determination of GDM risk.

## Conclusion

During the last decade, important progress has been made in the identification of GRS for the prediction of disease risk incl. T2DM. Our results utilising population-specific associated T2DM variants confirm that the genetic risk score can be derived successfully to identify females at increased risk of GDM in Central European populations. Whether GRS becomes a routine clinical tool for risk stratification of pregnant women (beyond traditional clinical risk factors) adopted by health care regulators and providers depends on several important factors. First of all, large-scale validation is necessary to prove the capacity to improve therapeutic decisions and health outcomes (which is certainly the case of GDM affecting both mother and offspring), but also on absolute risk of disease, diagnostic infrastructure, economic benefits and several other important aspects as recently comprehensively reviewed^31^.

## Supplementary Information

Supplementary Information 1.

Supplementary Information 2.

## Data Availability

The datasets generated during the study are not publicly available due to: (i) their sensitive nature (GDPR) and the potential risk of re-identification of subjects; (ii) the ethics committee did not approve the release of individual genetic data; (iii) the subjects did not consent to the disclosure of their genetic test results in individual personalised form, only in summary form, following statistical evaluation of the data. However, data can be obtained from the corresponding author for collaboration research purposes under the above conditions.
