# Supplementary figures and images for: Genetic risk score as a predictor of gestational diabetes in Central European Caucasians
Source: Sci Rep. 2026 Apr 29;16:20024. doi: 10.1038/s41598-026-49602-z (PMC13319762; doi:10.1038/s41598-026-49602-z)

## Slide 1
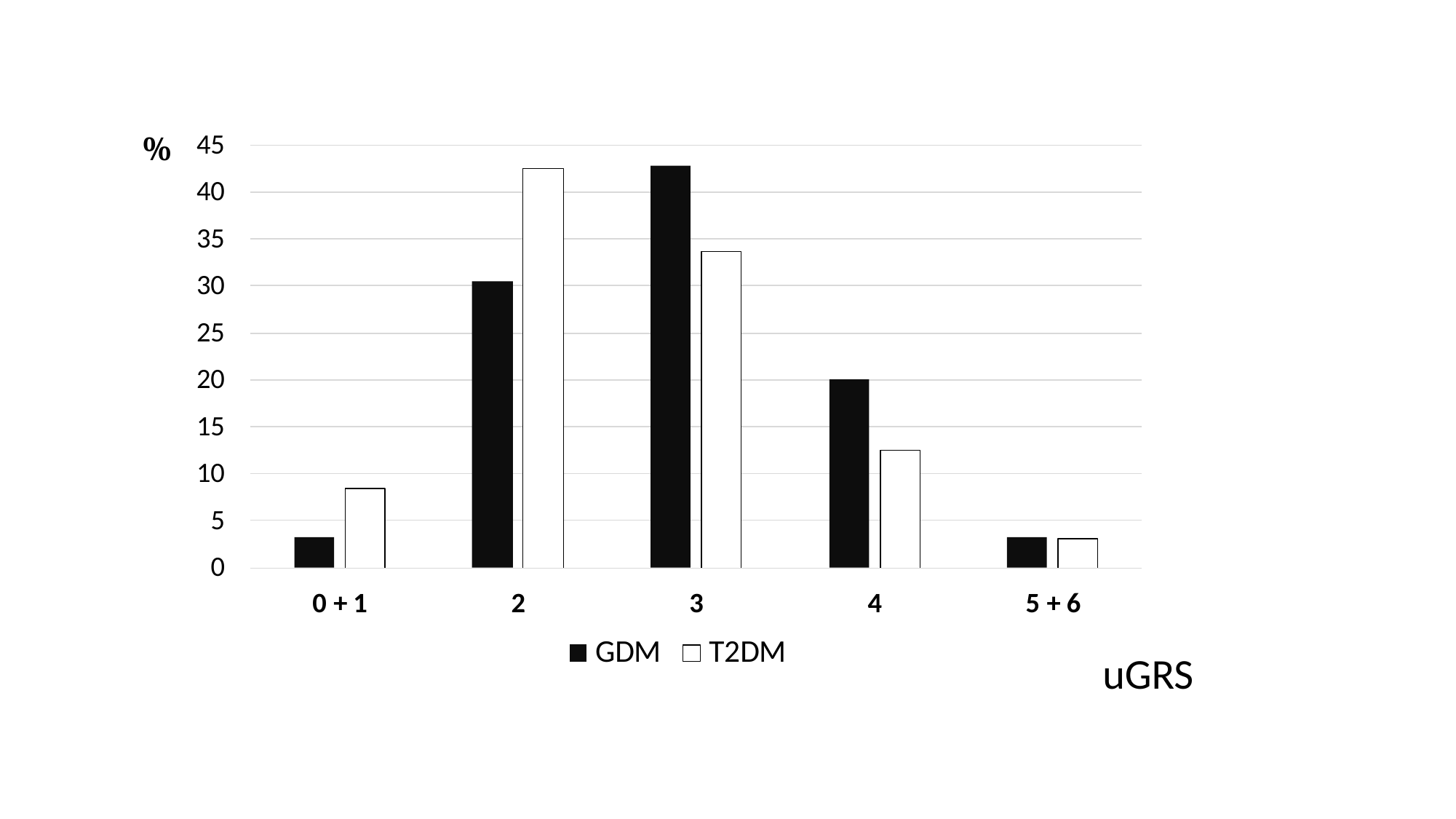

%
uGRS

Supplement: Supplementary file 1 — Supplementary Information 1. [file 41598_2026_49602_MOESM1_ESM.pptx]
