# Supplementary material for: Genetic risk score as a predictor of gestational diabetes in Central European Caucasians
Source: Sci Rep. 2026 Apr 29;16:20024. doi: 10.1038/s41598-026-49602-z (PMC13319762; doi:10.1038/s41598-026-49602-z)
Supplement: Supplementary file 2 — Supplementary Information 2. [file 41598_2026_49602_MOESM2_ESM.docx]

***Supplementary Table 1*.** List of the SNPs, examined in patients and controls.

| **SNP** | **Gene** | **TaqMan Assay ID** | **Nucleotide exchange** |
| --- | --- | --- | --- |
| rs10923931 | *NOTCH2* | C_1188816_10 | T/G |
| rs10203174 | *THADA* | C_29969028_10 | C/T |
| rs2943640 | *IRS1* | C_15949827_10 | C/A |
| rs4402960 | *IGF2BP2* | C_2165199_10 | T/G |
| rs6819243 | *MAEA* | C_30956854_10 | T/C |
| rs7756992 | *CDKAL1* | C_2504058_20 | G/A |
| rs849135 | *JAZF1* | C_7601134_10 | G/A |
| rs7845219 | *TP53INP1* | C_28987886_20 | T/C |
| rs10811661 | *CDKN2A/B* | C_31288917_10 | T/C |
| rs17791513 | *TLE4* | C_33884783_10 | A/G |
| rs12571751 | *ZMIZ1* | C_3154913_10 | A/G |
| rs7903146 | *TCF7L2* | C_29347861_10 | T/C |
| rs163184 | *KCNQ1* | C_3075708_20 | G/T |
| rs1552224 | *ARAP1* | C_1953903_10 | A/C |
| rsrs9936385 | *FTO* | C_34511515_10 | C/T |
| rs1801282 | *PPARG* | C_1129864_10 | C/G |
| rs11717195 | *ADCY5* | C_3035703_20 | T/C |
| rs4458523 | *WFS1* | C_32305990_20 | G/T |
| rs3802177 | *SLC30A8* | C_357887_10 | G/A |
| rs5215 | *KCNJ11* | C_2991148_10 | C/T |
| rs10830963 | *MTNR1B* | C_3256858_10 | G/C |

***Supplementary Table 2.*** Genotype distributions within examined subject.

M – major allele; m – minor allele

| **Gene** | **SNP**  **alleles** | **Group** | **MM** | | **Mm** | | **mm** | | **P** |
| --- | --- | --- | --- | --- | --- | --- | --- | --- | --- |
|  |  |  | N | % | N | % | N | % |  |
| ***FTO*** | rs9936385 | Controls | 388 | 33.1 | 561 | 47.9 | 221 | 18.9 | 0.19^#^ |
|  | M – T | GDM | 131 | 31.5 | 189 | 45.4 | 96 | 23.1 | 0.01^&^ |
|  | m – C | T2DM | 95 | 26.5 | 172 | 47.9 | 92 | 25.6 | 0.30^$^ |
| *TLE4* | rs17791513 | Controls | 1057 | 90.3 | 108 | 9.2 | 5 | 0.4 | 0.70^#^ |
|  | M – A | GDM | 373 | 89.7 | 41 | 9.9 | 2 | 0.5 | 0.79^&^ |
|  | m – G | T2DM | 326 | 90.8 | 31 | 8.6 | 2 | 0.6 | 0.59^$^ |
| *ADCY5* | rs11717195 | Controls | 757 | 64.7 | 367 | 31.4 | 46 | 3.9 | 0.13^#^ |
|  | M – T | GDM | 286 | 68.8 | 112 | 26.9 | 18 | 4.3 | 0.30^&^ |
|  | m – C | T2DM | 243 | 67.7 | 100 | 27.9 | 16 | 4.4 | 0.75^$^ |
| *CDKN2A/B* | rs10811661 | Controls | 792 | 67.7 | 343 | 29.3 | 35 | 3.0 | 0.50^#^ |
|  | M – T | GDM | 274 | 65.9 | 133 | 32.0 | 9 | 2.2 | 0.11^&^ |
|  | m – C | T2DM | 259 | 72.1 | 96 | 26.7 | 4 | 1.1 | 0.06^$^ |
| *IGF2BP2* | rs4402960 | Controls | 649 | 55.4 | 420 | 36.0 | 101 | 8.6 | 0.11^#^ |
|  | M – G | GDM | 208 | 50.0 | 173 | 41.6 | 35 | 8.4 | 0.08^&^ |
|  | m – T | T2DM | 175 | 48.7 | 148 | 41.2 | 36 | 10.0 | 0.74^$^ |
| ***TCF7L2*** | rs7903146 | Controls | 605 | 51.7 | 498 | 42.6 | 67 | 5.7 | 0.01^#^ |
|  | M – C | GDM | 185 | 44.5 | 195 | 46.9 | 36 | 8.7 | 0.00^&^ |
|  | m – T | T2DM | 150 | 41.8 | 157 | 43.7 | 52 | 14.5 | 0.04^$^ |
| *MAEA* | rs6819243 | Controls | 1105 | 94.4 | 63 | 5.4 | 2 | 0.2 | 0.75^#^ |
|  | M – T | GDM | 391 | 94.0 | 21 | 5.1 | 4 | 1.0 | 0.83^&^ |
|  | m – C | T2DM | 338 | 94.2 | 21 | 5.8 | 0 | 0.0 | 0.93^$^ |
| ***IRS1*** | rs2943640 | Controls | 429 | 36.7 | 577 | 49.3 | 164 | 14.0 | 0.01^#^ |
|  | M – C | GDM | 190 | 45.7 | 177 | 42.5 | 49 | 11.8 | 0.67^&^ |
|  | m – A | T2DM | 141 | 39.3 | 169 | 47.1 | 49 | 13.6 | 0.20^$^ |
| ***CDKAL1*** | rs7756992 | Controls | 601 | 51.4 | 491 | 42.0 | 78 | 6.7 | 0.01^#^ |
|  | M – A | GDM | 187 | 45.0 | 186 | 44.7 | 43 | 10.3 | 0.01^&^ |
|  | m – G | T2DM | 167 | 46.5 | 149 | 41.2 | 43 | 12.2 | 0.60^$^ |
| *THADA* | rs10203174 | Controls | 907 | 77.5 | 248 | 21.2 | 15 | 1.3 | 0.39^#^ |
|  | M – C | GDM | 331 | 79.6 | 82 | 19.7 | 3 | 0.7 | 0.06^&^ |
|  | m – T | T2DM | 295 | 82.2 | 62 | 17.3 | 2 | 0.6 | 0.36^$^ |
| *JAZF1* | rs849135 | Controls | 348 | 29.7 | 563 | 48.1 | 259 | 22.1 | 0.38^#^ |
|  | M – G | GDM | 112 | 26.9 | 200 | 48.1 | 104 | 25.0 | 0.92^&^ |
|  | m – A | T2DM | 104 | 29.0 | 177 | 49.3 | 78 | 21.7 | 0.54^$^ |
| ***ARAP1*** | rs1552224 | Controls | 815 | 69.7 | 321 | 27.4 | 34 | 2.9 | 0.00^#^ |
|  | M – A | GDM | 363 | 87.3 | 48 | 11.5 | 5 | 1.2 | 0.00^&^ |
|  | m – C | T2DM | 280 | 78.0 | 73 | 20.3 | 6 | 1.7 | 0.00^$^ |
| *KCNJ11* | rs5215 | Controls | 461 | 39.4 | 527 | 45.0 | 182 | 15.6 | 0.01^#^ |
|  | M – T | GDM | 134 | 32.2 | 222 | 53.4 | 60 | 14.4 | 0.76^&^ |
|  | m – C | T2DM | 134 | 37.3 | 169 | 47.1 | 56 | 15.6 | 0.21^$^ |
| *ZMIZ1* | rs12571751 | Controls | 319 | 27.3 | 582 | 49.7 | 269 | 23.0 | 0.87^#^ |
|  | M – A | GDM | 111 | 26.7 | 213 | 51.2 | 92 | 22.1 | 0.61^&^ |
|  | m – G | T2DM | 94 | 26.2 | 189 | 52.6 | 76 | 21.2 | 0.25^$^ |
| *PPARG* | rs1801282 | Controls | 826 | 70.6 | 305 | 26.1 | 39 | 3.3 | 0.70^#^ |
|  | M – C | GDM | 298 | 71.6 | 102 | 24.5 | 16 | 3.8 | 0.11^&^ |
|  | m – G | T2DM | 269 | 74.9 | 81 | 22.6 | 9 | 2.5 | 0.30^$^ |
| *WFS1* | rs4458523 | Controls | 391 | 33.4 | 564 | 48.2 | 215 | 18.3 | 0.07^#^ |
|  | M – G | GDM | 119 | 28.6 | 228 | 54.8 | 69 | 16.6 | 0.35^&^ |
|  | m – T | T2DM | 124 | 34.5 | 181 | 50.4 | 54 | 15.0 | 0.21^$^ |
| ***NOTCH2*** | rs10923931 | Controls | 914 | 78.1 | 228 | 19.5 | 28 | 2.4 | 0.05^#^ |
|  | M – G | GDM | 305 | 73.3 | 111 | 26.7 | 0 | 0.0 | 0.28^&^ |
|  | m – T | T2DM | 290 | 80.8 | 65 | 18.1 | 4 | 1.1 | 0.01^$^ |
| *TP53INP1* | rs7845219 | Controls | 329 | 28.1 | 600 | 51.3 | 241 | 20.6 | 0.06^#^ |
|  | M – T | GDM | 115 | 27.6 | 193 | 46.4 | 108 | 26.0 | 0.00^&^ |
|  | m – C | T2DM | 82 | 22.8 | 170 | 47.4 | 107 | 29.8 | 0.24^$^ |
| *KCNQ1* | rs163184 | Controls | 318 | 27.2 | 574 | 49.0 | 278 | 23.8 | 0.13^#^ |
|  | M – G | GDM | 96 | 23.1 | 204 | 49.0 | 116 | 27.8 | 0.26^&^ |
|  | m – T | T2DM | 82 | 22.8 | 185 | 51.5 | 92 | 25.6 | 0.73^$^ |
| *SLC30A8* | rs3802177 | Controls | 574 | 49.1 | 495 | 42.3 | 101 | 8.6 | 0.26^#^ |
|  | M – G | GDM | 197 | 47.4 | 192 | 46.2 | 27 | 6.5 | 0.18^&^ |
|  | m – A | T2DM | 196 | 54.6 | 137 | 38.2 | 26 | 7.2 | 0.08^$^ |
| ***MTNR1B*** | rs10830963 | Controls | 595 | 50.9 | 463 | 39.6 | 112 | 9.6 | 0.00^#^ |
|  | M – C | GDM | 153 | 36.8 | 210 | 50.5 | 53 | 12.7 | 0.65^&^ |
|  | m – G | T2DM | 173 | 48.2 | 148 | 41.2 | 38 | 10.6 | 0.01^$^ |

Genes named in bold have been included into the GRS calculations.

P values are given for comparisons as follows:

GDM vs. Controls^#^

Controls vs. T2DM^&^

GDM vs. T2DM^$^

**Supplementary Table 3**.

Individual variants significantly associated with GDM in cross-validation subsets for control females vs GDM.

|  |  |  | Subset 1 |  | Subset 2 |  | Subset 3 |  |
| --- | --- | --- | --- | --- | --- | --- | --- | --- |
| Gene | SNP | Calculated for | OR (95 % CI) | P | OR (95 % CI) | P | OR (95 % CI) | P |
| *TCF7L2* | rs7903146 | +T vs. CC | 1.34 (0.90 - 2.00) | 0.150 | 1.25 (0.85 - 1.84) | 0.248 | 1.43 (0.97 - 2.10) | 0.072 |
| *IRS1* | rs2943640 | CC vs. +A | 1.73 (1.16 - 2.59) | 0.007 | 1.51 (1.02 - 2.22) | 0.039 | 1.19 (0.80 - 1.75) | 0.392 |
| *CDKAL1* | rs7756992 | +G vs. AA | 1.22 (0.82 - 1.82) | 0.321 | 1.16 (0.79 - 1.71) | 0.441 | 1.45 (0.99 - 2.13) | 0.057 |
| *ARAP1* | rs1552224 | AA vs. +C | 3.62 (2.00 - 6.55) | <0.001 | 3.02 (1.78 - 5.12) | <0.001 | 2.52 (1.50 - 4.24) | <0.001 |
| *NOTCH2* | rs10923931 | +T vs. GG | 1.57 (1.00 - 2.46) | 0.052 | 1.27 (0.82 - 1.97) | 0.285 | 1.11 (0.71 - 1.74) | 0.653 |
| *MNTR1B* | rs10830963 | +G vs. CC | 1.82 (1.21 - 2.74) | 0.004 | 1.86 (1.25 - 2.76) | 0.002 | 1.68 (1.13 - 2.48) | 0.010 |
| *FTO* | rs9936385 | CC vs. +T | 1.00 (0.60 - 1.65) | 0.998 | 1.63 (1.03 - 2.59) | 0.038 | 1.52 (0.97 - 2.37) | 0.069 |
